# Short-term rehospitalisation or death and determinants after admission for acute heart failure in a cohort of African patients in Port Harcourt, southern Nigeria

**DOI:** 10.5830/CVJA-2017-038

**Published:** 2018

**Authors:** R Akpa Maclean, Iheji Okechukwu

**Affiliations:** Cardiovascular Division, Department of Internal Medicine, Faculty of Clinical Sciences, University of Port Harcourt, Port Harcourt, Nigeria; Cardiovascular Division, Department of Internal Medicine, University of Port Harcourt Teaching Hospital, Port Harcourt, Nigeria

**Keywords:** heart failure, outcomes, rehospitalisation, mortality

## Abstract

**Background:**

Heart failure (HF) is a major health burden globally and contributes significantly to morbidity and mortality related to cardiovascular disease. The aim of this study was to determine the outcome, and factors determining these outcomes in patients admitted for acute HF and followed up for six months.

**Methods:**

This was a hospital-based, prospective study. Subjects included consecutive patients with a confirmed diagnosis of acute HF admitted to the medical wards of the University of Port Harcourt Teaching Hospital (UPTH) in Nigeria over one year. All had a full physical examination and relevant investigations, including echocardiography. Subjects were followed up for six months and reassessed for outcome/ endpoint, which was rehospitalisation or death. Factors that predicted these outcomes were also determined.

**Results:**

There were 160 subjects, 84 females and 76 males, age range 20 to 87 years, mean age 52.49 ± 13.89 years. Sixteen subjects (10.0%) were lost to follow up, 66 (41.3%) showed clinical improvement, 57 (35.6%) were rehospitalised, while 21 (13.1%) died. Determinants of rehospitalisation were New York Heart Association (NYHA) class, heart failure type, haemoglobin level at presentation and estimated glomerular filtration rate (eGFR). Determinants of mortality were NYHA class and haemoglobin level at presentation.

**Conclusion:**

Heart failure rehospitalisation and mortality rates of 35.6 and 13.1%, respectively, were high compared to developed countries.

Heart failure (HF) is the end stage of most diseases of the heart and a major cause of morbidity and mortality. Thomas Lewis aptly captured the high premium placed on HF as far back as 1933 when he remarked, ‘The very essence of cardiovascular practice is the early detection of heart failure’.^1^ The worldwide prevalence and incidence rates of HF are approaching epidemic levels, as evidenced by the increasing number of HF hospitalisations and HF-attributable mortalities, as well as the high costs associated with the care of HF patients.^2^

Worldwide, HF affects almost 23 million people,^2^ with nearly five million people in the United States3 and up to three million people in the United Kingdom being affected.^4^ It is estimated to account for about 5% of admissions to hospital medical wards, with over 100 000 annual admissions in the United Kingdom.^1^ The financial burden of HF in most countries is very substantial. In the United States about $37.2 billion was spent directly or indirectly on HF management in 2009, with $20.1 billion of the expenditure largely related to hospitalisation.^5^

In Africa, HF has become a dominant form of cardiovascular disease, with great social and economic consequences due to its high prevalence and mortality rate, and the impact on young, economically active individuals.^5^ The peak incidence of HF in African patients remains in the fifth decade,^6^ and hospital case fatality rates range from nine to 12.5%.^7^ This high death rate ranks HF among the major causes of death of cardiovascular origin in Africa.^7^ In Port Harcourt, Niger delta region of Nigeria, HF was the third commonest non-communicable cause of admission (next to diabetes and its complications and cerebrovascular disease) and contributed 9.6% of patients admitted to the medical wards over a five-year period.^8^,^9^

The prognosis of HF is uniformly poor. The one-year mortality rate in patients with severe HF (NYHA class IV) is between 30 and 70%, and in patients with HF in NYHA classes I–III, the annual mortality rate is five to 10%.^10^,^11^ Other important variables that have been found to influence the outcome in HF patients include co-morbidities, estimated glomerular filtration rate (eGFR) and haemoglobin level, left ventricular function, as well as treatment or interventions received.^12^-^15^

Identifying the predictors of rehospitalisation and mortality among HF patients is vital in helping physicians to risk stratify their HF patients and chart the best possible post-discharge plan.^16^ There is however a dearth of data on the outcome profile of patients admitted with HF in the Niger delta region of Nigeria. The aim of this study was to determine the short-term (six-month) outcome and factors influencing these outcomes in patients admitted with acute HF in Port Harcourt, southern Nigeria.

## Methods

The was a hospital-based, prospective study carried out in the medical wards of the University of Port Harcourt Teaching Hospital (UPTH), Port Harcourt, Niger delta region of Nigeria. All the patients admitted to the medical wards with a confirmed diagnosis of acute heart failure (AHF) from 1 January to 31 December 2014 were recruited. The patients were selected if they met the Framingham clinical criteria^17^ for the diagnosis of HF and confirmed on echocardiography.

Demographic data were obtained from all patients aged 18 years and older who gave written, informed consent. The hospital’s ethics committee approved the study. The NYHA functional class, and baseline clinical and demographic characteristics of patients were obtained using a structured questionnaire. All study subjects underwent full clinical examinations, anthropometric measurements and relevant investigations, including chest radiography, electrocardiogram and echocardiogram.

Blood pressure was measured with a standard mercury sphygmomanometer (cuff size 12.5 × 40 cm) using standard protocols. Systolic and diastolic blood pressures were taken at Korotkoff phases 1 and 5, respectively, to the nearest 2 mmHg.^18^ Hypertension was deemed present if systolic blood pressure was 140 mmHg or above and/or diastolic blood pressure was 90 mmHg or above on at least two occasions, or if the patient was receiving anti-hypertensive drug treatment.^18^

Waist circumference was measured in centimetres at the midpoint between the lower costal margin and the iliac crest, with the patient standing and the feet positioned close together. The value was read at the end of a normal expiration.^19^ Waist circumference was considered increased if greater than 88 cm in women and 102 cm in men.^19^ Hip circumference was measured similarly but at the level of the greater trochanter. Waist–hip ratio was calculated using the formula: waist (cm)/hip (cm).^19^ Weight was measured with a mechanical weighing scale with the subject wearing only light clothing, and height was measured using a stadiometer with the subject standing with feet together, without shoes or head gear. The reading was taken to the nearest 0.5 cm.

Body mass index (BMI) was calculated using the formula weight (kg)/height^2^ (m). BMI status was classified according to the WHO criteria as normal weight (18.5–24.9 kg/m^2^), overweight (25–29.9 kg/m^2^), class I obesity (30.0–34.9 kg/m^2^), class II obesity (35.0–39.9 kg/m^2^), and morbid obesity (≥ 40 kg/m^2^).^19^

Blood samples were collected from all patients and analysed for haemoglobin level, fasting lipid profile, and serum urea, creatinine and plasma glucose levels. Serum creatinine level was used to calculate the eGFR with the Cockcroft–Gault formula.^20^ Severity of renal impairment was classified using the National Kidney Foundation-developed criteria as part of its Kidney Disease Outcomes Quality Initiative (NKF KDOQI) to stratify chronic kidney injury.^21^

Fasting serum cholesterol and triglyceride levels were measured using the enzymatic method with a reagent from Atlas Medical Laboratories. Fasting high-density lipoprotein (HDL) cholesterol was measured with the precipitation method. Low-density lipoprotein (LDL) cholesterol values were calculated using the Friedwald equation when the triglyceride level was less than 4.0 mmol/l: LDL = TC – (HDL + TG /2.2).^22^

Standard 12-lead elctrocardiography was performed for all patients and the parameters assessed included presence of atrial fibrillation, pathological Q waves, left ventricular hypertrophy, QT prolongation and ST abnormalities. Transthoracic echocardiography was performed on all the subjects and assessments were done according to the recommendations of the American Society of Echocardiography.^23^

Left ventricular (LV) systolic performance was assessed using fractional shortening (FS) and the ejection fraction (EF) of the left ventricle. These were calculated automatically by the machine using the Teichoiz formula.^24^ Left ventricular mass (LVM) was calculated using the American Society of Echocardiography recommended formula for estimation of LV mass from LV linear dimensions.^25^ Left ventricular mass index (LVMI) was calculated by indexing the LVM to the body surface area. Left ventricular hypertrophy (LVH) was defined in absolute terms as LVMI > 115 g/m^2^ in men and > 95 g/m^2^ in women.^25^ LV diastolic function was evaluated by studying the filling dynamics of the left ventricle, the isovolumetric relaxation time (IVRT), pulmonary venous flow and tissue Doppler imaging-derived myocardial wall velocities.^26^

All the study patients were followed up for six months or until death if the patient died before six months of follow up. They were assessed during follow up by telephone contacts if they did not keep out-patient appointments. The primary endpoints were death due to any cause and rehospitalisation. The duration of follow up was defined as the interval from the date of the index examination at which the echocardiogram was obtained to the date of death or the date of last contact. During six months of follow up, clinical and echocardiographic parameters were obtained and compared with initial values.

## Statistical analysis

Data were analysed using the Statistical Package for Social Sciences (SPSS) version 20.0. Results are presented as mean ± standard deviation for continuous variables, while categorical variables are expressed as proportions or percentages. Tables are used to illustrate results where appropriate. Continuous variables were compared by the Student’s t-test, while proportions or categorical parameters were compared with the chi-squared test or two-tailed Fisher’s exact test, as appropriate. Logistic regression analysis was done where appropriate. A p-value of less than 0.05 was considered statistically significant.

## Results

A total of 160 patients, 84 females and 76 males, were studied over the study period. The age range was 20 to 87 years with a mean age of 52.49 ± 13.89 years. A total of 16 subjects (10%) were lost to follow up, 66 subjects (41.3%) improved clinically and continued their regular out-patient clinic attendance for six months, 57 subjects (35.6%) were rehospitalised for worsening of HF symptoms, while 21 subjects (13.1%) died.

The socio-demographic profile of the patients did not have any significant effect on rehospitalisation and mortality. There was a significant association between rehospitalisation and NYHA class, type of HF (systolic or diastolic HF), BMI, haemoglobin level, LVEF and eGFR (Table 1). However, when the effects of confounding variables were removed using the logistic regression model, the real determinants of rehospitalisation were NYHA class, type of heart failure, haemoglobin level and eGFR (Table 2). There was a significant association between mortality and NYHA class, haemoglobin level and LVEF (Table 3). However after logistic regression analysis, only NYHA class and haemoglobin level at presentation were the real determinants of mortality (Table 4).

**Table 1:** Association of different variables with rehospitalisation

	*Rehospitalisation*	
*Variable*	*No* *(%)*	*Yes* *n (%)*	*Total* *Variable n*	*Chi2 (p-value)*
Gender				1.033 (0.309)
Male	52 (68.4)	24 (31.6)	76	
Female	51 (60.7)	33 (39.3)	84	
Age group (years)				4.95 (0.084)
18–45	26 (52.0)	24 (48.0)	50	
45–65	56 (70.9)	23 (29.1)	79	
> 65	21 (67.7)	10 (32.3)	31	
Level of education				0.24 (0.623)
None/primary	29 (67.4)	14 (32.6)	43	
Secondary/tertiary	74 (63.2)	43 (36.8)	117	
NYHA class at Presentation				26.64 (< 0.001)^*^
Class II	39 (90.7)	4 (9.3)	43	
Class III	44 (47.8)	48 (52.2)	92	
Class IV	20 (80.0)	5 (20.0)	25	
Type of HF				6.05 (0.014)^*^
Diastolic HF	14 (93.3)	1 (6.7)	15	
Systolic HF	89 (61.4)	56 (38.6)	145	
BMI (kg/m2)				11.72 (0.003)^*^
≤ 24.99	34 (64.2)	19 (35.8)	53	
25–29.99	35 (52.2)	32 (47.8)	67	
≥ 30	34 (85.0)	6 (15.0)	40	
Haemoglobin (g/dl)				5.51 (0.019)^*^
≥ 10	84 (69.4)	37 (30.6)	121	
< 10	19 (48.7)	20 (51.3)	39	
LVEF (%)				7.52 (0.023)^*^
≥ 40	48 (77.4)	14 (22.6)	62	
25–39.99	40 (56.3)	31 (43.7)	71	
< 25	15 (55.6)	12 (44.4)	27	
eGFR (ml/min)				11.17 (0.001)^*^
≥ 60	76 (73.8)	27 (26.2)	103	
< 60	27 (47.4)	30 (52.6)	57	

NYHA = New York Heart Association; BMI = body mass index; LVEF = left ventricular ejection fraction; eGFR = estimated glomerular filteration rate; n = number; % = percentage within variable; ^*^significant p-value.

**Table 2:** Result of logistic regression analysis of some variables with rehospitalisation

*Variable*	*B*	*P*	*R2*
NYHA class			0.296
Class II	–	< 0.001	
Class III	1.022	0.271	
Class IV	2.819	< 0.001^*^	
Type of HF	2.711	0.032^*^	
BMI (kg/m2)			
< 24.99	–	0.410	
25–29.99	0.158	0.812	
≥ 30	0.635	0.285	
Haemoglobin			
< 10 g/dl	1.432	0.012^*^	
LVEF (%)			
≥ 40	–	0.475	
25–39.99	–0.879	0.225	
< 25	–0.461	0.435	
eGFR < 60 ml/min	1.085	0.024^*^	

NYHA = New York Heart Association; BMI = body mass index; LVEF = left ventricular ejection fraction; eGFR = estimated glomerular filteration rate; ^*^significant p-value.

**Table 3:** Association of some variables with mortality

	*Mortality*		*Total* *No*
*Variables*	*No, n (%)*	*Yes, n (%)*		*Chi2 (p-value)*
Gender
Male	63 (82.9)	13(17.1)	76	2.01 (0.156)
Female	76 (90.5)	8 (9.5)	84	
Age group (years)
18–45	43 (86.0)	7 (14.0)	50	3.724 (0.155)
46–65	72 (91.1)	7 (89)	79	
> 65	24 (77.4)	7 (22.6)	31	
Level of education
Nursery/primary	36 (95.3)	7 (16.3)	43	0.513 (0.474)
Secondary/tertiary	103 (88.0)	14 (12.0)	117	
NYHA class
Class II	41 (95.3)	2 (4.7)	43	57.10 (< 0.001)^*^
Class III	88 (95.7)	4 (4.3)	92	
Class IV	10 (40.0)	15 (60.0)	25	
Type of HF
Diastolic HF	15 (100.0)	0 (0.0)	15	2.501 (0.114)
Systolic HF	124 (85.5)	21 (14.5)	145	
BMI (kg|m2 )
≤ 24.99	45 (84.9)	8 (15.1)	53	5.49 (0.064)
25–29.99	55 (82.1)	12 (17.9)	67
≥ 30	39 (97.5)	1 (2.5)	40
Haemoglobin (g/dl)
≥ 10	110 (90.9)	11 (9.1)	121	7.09 (0.008)^*^
< 10	29 (74.4)	10 (25.6)	39	
LVEF (%)
≥ 40	60 (96.8)	2 (3.2)	62	8.72 (0.013)^*^
25–39.99	57 (80.3)	14 (19.7)	71	
< 25	22 (81.5)	5 (18.5)	27	
eGFR (ml/min)
≥ 60	93 (90.3)	10 (9.7)	103	2.96 (0.085)
< 60	46 (80.7)	11 (19.3)	57

NYHA = New York Heart Association; BMI = body mass index; LVEF = left ventricular ejection fraction; eGFR = estimated glomerular filteration rate; n = number; % = percentage within variable; ^*^significant p-value.

**Table 4:** Logistic regression analysis of some variables with mortality

*Variable*	*B*	*p-value*	*R2*
NYHA class			0.284
Class II		< 0.001
Class III	–3.96	0.001^*^
Class IV	–4.76	< 0.001^*^
Haemoglobin (g/dl)	0.048	0.950
LVEF (%)
≥ 40		0.018
25–39.99	0.587	0.643
< 25	2.682	0.014^*^

NYHA = New York Heart Association; LVEF = left ventricular ejection fraction; ^*^significant p-value.

## Discussion

The average age of the HF patients in this study was 52.49 ± 13.89 years, which is similar to the pattern seen in other African countries but at variance with that of patients in Western countries where HF remains predominantly a disease of the elderly.^27^,^28^ In Spain, Permanyer et al.^29^ found that almost 40% of HF patients were over 80 years and more than 70% were over 70 years. This is also the pattern in the United States of America where average age was about 70 years for HF patients.^30^

The lower average age in our study and that of other studies emanating from Africa may be attributable to the fact that the major causes of HF in sub-Saharan Africa, such as hypertension, rheumatic heart disease, idiopathic dilated cardiomyopathy and HIV-related heart disease affect mainly young and middle-aged people.^31^,^32^ Also hypertension detection, treatment and control in Nigeria, as in other African countries, is poor and complications such as heart failure is expected to occur earlier. The major aetiologies of heart failure in this study were hypertension, dilated cardiomyopathy and rheumatic valve disease, which is in keeping with studies from other parts of Africa.^31^,^32^

Late presentation of patients to hospital was a significant finding in this study; 57.5% of the patients presented in NYHA class III and 15.6% in class IV. This late presentation is similar to findings from other studies documented by investigators on the African continent.^5^,^15^,^33^ Presentation in an advanced NYHA class impacts negatively on the prognosis and outcome of heart failure patients.^15^ This fact was also shown in our study where the severity of the NYHA functional class of the patient at presentation was found to be a determinant of outcome.

In this study, 10% of the patients were lost to follow up and all attempts at location were futile. The reasons were not clear. The rehospitalisation rate for HF in this study was 35.6%, which is higher than figures from the United States where Ross et al.,^34^ using data from the Medicare, documented 30-day re-admission rates after HF hospitalisation of 23.0% in 2004, 23.3% in 2005 and 22.9% in 2006. Therefore hospitalisation and rehospitalisation of HF patients continues to be a great public heath burden, especially in a developing economy such as Nigeria.

The mortality rate of 13.1% in this study is similar to the figures documented for hypertensive HF patients in the same institution about two decades ago, where investigators reported a mortality rate of 13.6%.^35^ This finding suggests that mortality rate from HF in our environment has remained relatively stable despite advances in treatment modalities. This rate is also comparable to the mortality rate of 10% reported in the northern part of Nigeria.^36^ It is however much lower than the 30.8% documented from western Nigeria,^33^ and the 35% documented in Lusaka, Zambia.^37^

The high mortality rate from western Nigeria may be attributable to late presentation, with more than 90% of patients presenting in NYHA class IV, whereas the Zambian investigators admitted logistic and financial challenges that made it difficult to optimise a patient’s treatment. However Ogah et el.^38^ in a recent study in the south-west region of Nigeria reported a rehospitalisation rate of 12.2% and mortality rate of 4.2% at six months of follow up.

The determinants of rehospitalisation in this study wereNYHA class at presentation (higher NYHA class was associatedwith higher re-admission rate), type of heart failure (systolicheart failure), low haemoglobin level (< 10 g/dl) and low eGFR(< 60 ml/min), while the identified determinants or predictors ofsix-month mortality were high NYHA class (class III and IV)and low LVEF (< 25%). These findings agree with the results ofother studies done within and outside Africa.

Falase et al.^39^ reported the prognostic importance of anaemia in HF patients, Karaye et al.^36^ noted the poor prognostic value of low LVEF of < 40%, Familoni et al.^15^ reported factors associated with poor outcome in HF patients to include anaemia, low eGFR, increased age and low haemoglobin level of < 10 g/dl. Nohria et al.^3^ documented high NYHA class, low LVEF, advanced age, low eGFR, anaemia and other co-morbid conditions as factors that negatively affect outcome in HF patients.

Ogah et al.^38^ from south-western Nigeria however identified factors associated with six-month rehospitalisation to include presence of mitral regurgitation, age ≥ 60 years, presence of tricuspid regurgitation and atrial fibrillation, and LVEF. Using data from the sub-Saharan African Survey of Heart Failure (THESUS-HF), Sliwa et al.^40^ also noted that the main predictors of 60-day re-admission or death were a history of malignancy and severe lung disease, admission systolic blood pressure, heart rate and signs of congestion (rales), kidney dysfunction (BUN), anaemia, HIV positivity and echocardiographic ejection fraction.

The determinants of mortality in our study were similar to findings from other parts of Nigeria and sub-Saharan Africa. This is probably because the aetiology of heart failure in this region is similar and due mainly to hypertension, cardiomyopathy and rheumatic valvular heart disease.

## Conclusion

As in other studies of HF patients in sub-Saharan Africa, HF patients in the south-south region of Nigeria were relatively young, being in their fifth to sixth decades of life, and presented in advanced NYHA functional class. The determinants of mortality were high NYHA class, low eGFR and anaemia, while the determinants of rehospitalisation were anaemia, low LVEF, systolic heart failure and impaired renal function.
